# Patient Satisfaction With Public Pharmacy Services: Structural and Policy Implications From Greece

**DOI:** 10.7759/cureus.58654

**Published:** 2024-04-20

**Authors:** Stefanos Karakolias, Christina Georgi, Vasileios Georgis

**Affiliations:** 1 Post-Graduate Program in Health Care Management, Hellenic Open University, Patras, GRC; 2 Business Administration Department, University of Patras, Patras, GRC

**Keywords:** greece, medicinal advise, high-cost drugs, eopyy pharmacies, public pharmacies, patient satisfaction

## Abstract

Objectives

This study aimed at investigating patient satisfaction with services offered by a certain type of public pharmacies in Greece (National Organisation for Healthcare Provision (EOPYY) pharmacies), tasked with dispensing mostly high-cost drugs, in an effort to highlight the aspects to be optimized.

Methods

The Patient Satisfaction with Pharmacist Services Questionnaire 2.0 (PSPSQ 2.0) questionnaire was the main instrument of our research. We received 201 full responses from patients themselves and patients' companions who had visited EOPYY pharmacies in Athens, the capital city of Greece, from October 2022 to January 2023.

Results

Patients seem satisfied with public pharmacies in general. In fact, the professionalism of the pharmacists, the respect that patients have received from them, and the information and explanations that were given by pharmacists, received a very high score. On the other hand, the parameters referred to the information that patients received from pharmacists for the overall improvement of their health had the lowest score, revealing an apparent lag in the field of medicinal advice.

Conclusion

Without any doubt, patients expect their pharmacists to be more guiding and to better communicate this role. This requires more time to be spent with patients, focused training, teamwork, layout, and other organizational interventions.

## Introduction

Pharmacies are primarily charged with dispensing medicinal products, but in recent years, they have acquired the upgraded role of providing more comprehensive care, including health promotion, education, and prevention [1,2]. Since pharmacies are on the frontline of primary care and the overall health system, it is crucial to fulfill users’ expectations as reflected by their satisfaction levels [3]. Satisfaction (or dissatisfaction) is the result of users’ evaluation of both the service offered by the pharmacists and the interaction with them [4,5]. Previous research showed that patient satisfaction positively affects patient adherence to the treatment [6] and improves health outcomes [7]. As such, optimizing patient satisfaction seems a key driver toward improving pharmaceutical services.

Public pharmacies operate supplementary to private ones, across various health systems, for dispensing purposes. In the Greek case, public pharmacies are those being run by either public hospitals or the National Organisation for Healthcare Provision (EOPYY), namely, the Unified Healthcare Fund, which, in 2012, turned the market into a near monopsony. The EOPYY is the major buyer of health services and medicines, setting the conditions required to purchase products with greater discounts and creating sufficient conditions for economies of scale, thus ensuring unhindered access to medicines, even in periods of financial crisis, as in 2012 [8-10]. The latter ones, hereinafter “EOPYY pharmacies”, include 35 units throughout the Greek territory, mainly tasked with dispensing, free of charge, high-cost drugs for chronic and/or severe diseases [11], including anti-cancer drugs, immunosuppressants, immunoglobulins, cardiac stimulants, etc. It is worth mentioning that EOPYY pharmacies are not the exclusive distributors of high-cost drugs since those intended for purely hospital use are dispensed mainly by public hospital pharmacies [12]. In this context, measuring patient satisfaction with such services, provided by the state itself for clinically special cases, offers a compelling insight into health policy orientation and effectiveness.

The Greek literature includes only a couple of studies on patient satisfaction with services offered by EOPYY pharmacies [13,14]. Actually, both of them include public hospitals’ pharmacies and focus on specific patient groups (rheumatoid arthritis and multiple sclerosis, respectively). Another fact is that the aforementioned surveys examine the EOPYY’s early years when the number of EOPYY pharmacies was much lower; thus, availability problems were mostly reported: long distances from EOPYY pharmacies and difficulties in obtaining the prescribed medicine (non-availability of it, longer waiting time, etc.). In any case, there is a complete absence of studies focused on this discrete type of public pharmacies and the full range of their patients.

Within this framework, our study aimed at investigating patient satisfaction with services offered by EOPYY pharmacies, in an effort to formulate improvement proposals based on international experience.

## Materials and methods

To proceed with this study, we first investigated the literature on the relationship’s development, especially the one that focuses on the relationship between pharmacists and patients. Based on the bibliographic search and the empirical knowledge acquired in the context of this research, we started by doing an exploratory study to determine both the extent of the questionnaire and whether the way questions were formulated was clear and comprehensible.

Initially, we approached five pharmacists currently working at EOPYY pharmacies and five random patients, and we asked them whether we could discuss the questionnaire with them. During this stage, two pharmacists and four patients responded and showed interest in the subject of the research. Thus, we perceived what concerned the patients, compared what the two sides believed, and decided on the extent of the questionnaire.

We used the Patient Satisfaction with Pharmacist Services Questionnaire 2.0 (PSPSQ 2.0) [15,16] as the main instrument of our research. A 5-point Likert scale was used for all our variables, starting from "strongly agree" to "strongly disagree". We submitted our questionnaire to the administration of the organization, and we were given permission to carry out the study but without personal patient information (approval number: 23350/16.09.2022). We also clarified that patient participation is voluntary.

Then, we addressed EOPYY pharmacies, located in the capital city of Greece, Athens, and approached patients who had come there to receive their medicine. They were asked randomly to participate in our research. After they had given their informed consent, the questionnaire was administered to them. Our research took place from October 2022 until January 2023. We approached 1,000 patients in total and received 201 questionnaires fully completed. It should be noted that, in addition to the patients themselves, we included patients' companions, who often procure the medicines for them, as many patients who address EOPYY pharmacies cannot go there themselves due to their poor health. The only inclusion criterion used in our research was to have been served personally at least once by EOPYY pharmacies.

Toward a better understanding of the respondents’ profile, the final sample included more women (58.7%), one-third of the respondents were between 36 and 45 years old, one-half of them were suffering from their illness for up to five years, and three-fourths of them were using the pharmacy service at least once every three months.

## Results

To examine the reliability of the factors included in our research, we relied on the analysis of means and standard deviation. The figures illustrated in Table 1 suggest that all factors approach a normal distribution, as mean and median values approach each other and the standard deviation value is rather low. The values of skewness and kurtosis range well below ±2.2, and in some cases close to zero, so we did not consider these values to be far from the normal distribution [17]. Considering both average values and confidence intervals (95%) in Table 1, it is also extracted that patients rated all aspects of EOPYY pharmacies highly, in general. All averages were clearly above 3, with a lower value of 3.24 received by the factor "The pharmacist gave useful recommendations regarding the management of my overall health", while the factor "The pharmacist was professional in all our interactions" received the highest score with a value of 4.22. The waiting time factor received a value of 3.72, while the overall satisfaction with EOPYY pharmacies received a value of 3.97. Even in the factor “The pharmacist gave useful recommendations about managing my overall health”, the lowest value of the confidence interval was 3.08, well above 2.5, that is, above average, and marginally above 3.

**Table 1:** Summary statistics of all variables used in the research

Factor	Average	Std. deviation	95% Confidence interval of the mean		Std. error of the mean	Median	Skewness	Kurtosis
			Low limit	High limit				
The pharmacist fully addressed my concerns and health issues during my visit	4.03	0.854	3.91	4.15	0.06	4.00	-0.641	0.082
The pharmacist was professional in all our interactions	4.22	0.771	4.12	4.33	0.054	4.00	-0.739	0.056
The pharmacist gave me information and explained in a way I could understand	4.15	0.835	4.03	4.27	0.059	4.00	-0.859	0.559
The pharmacist checked that I understood all the information	3.79	0.984	3.65	3.92	0.069	4.00	-0.639	-0.027
The pharmacist took as much time as necessary to help me with my questions and concerns	3.91	0.944	3.78	4.04	0.067	4.00	-0.755	-0.259
The pharmacist made sure I understood how important it is to follow the medication regimen	3.63	1.042	3.48	3.77	0.073	4.00	-0.356	-0.626
The pharmacist gave helpful recommendations on how to take my medication	3.55	1.170	3.38	3.71	0.083	4.00	-0.399	-0.822
The pharmacist gave helpful recommendations about managing my overall health	3.24	1.110	3.08	3.39	0.078	3.00	-0.086	-0.756
The pharmacist worked with me to manage medication-related issues	3.59	1.124	3.43	3.74	0.079	4.00	-0.497	-0.542
The pharmacist was kind and caring in dealing with my health issues	4.02	0.880	3.90	4.15	0.062	4.00	-0.849	0.648
The pharmacist encouraged me to achieve my treatment goals	3.37	1.160	3.21	3.53	0.082	3.00	-0.339	-0.698
I felt comfortable in my interactions with the pharmacist	3.99	0.875	3.86	4.11	0.062	4.00	-0.922	0.879
The pharmacist was respectful to me during our interactions	4.13	0.779	4.03	4.24	0.055	4.00	-0.944	1.365
The pharmacist is dedicated to improving my health	3.40	1.128	3.25	3.56	0.08	4.00	-0.337	-0.688
I could trust the information provided by the pharmacist	3.85	1.001	3.71	3.98	0.071	4.00	-0.865	0.586
I am satisfied with my waiting time	3.72	1.078	3.57	3.87	0.076	4.00	-0.707	-0.012
I am satisfied with the information I get	3.89	0.923	3.76	4.01	0.065	4.00	-0.734	0.187
I am satisfied with their professionalism	4.01	0.889	3.89	4.13	0.063	4.00	-0.926	0.778
I am satisfied with their abilities	3.95	0.928	3.82	4.07	0.065	4.00	-0.989	1.14
I am overall satisfied with the EOPYY pharmacy	3.97	0.995	3.83	4.11	0.07	4.00	-1.203	1.447

Next, we represent in Table 2 the percentages of patient responses by degree of agreement and factor. It is observed that the answers in all cases are shifted toward the positive side and the positive opinion always prevails. After having articulated these percentages in a negative opinion (disagree and strongly disagree), a neutral opinion (neither agree nor disagree), and a positive opinion (agree and strongly agree), the lowest level of satisfaction was obtained by the statement "The pharmacist gave me useful recommendations for managing my overall health" (41.8% of the respondents), while also low percentages were obtained by the factors "My pharmacist encouraged me to achieve my therapeutic goals" (49.7% of the respondents) and "The pharmacist is dedicated to improving my health" (51.2% of the respondents). As a result, these specific factors received high rates of dissatisfaction (i.e., 26.9%, 23.4%, and 22.9%, respectively). On the other side, patients seem quite happy with their waiting time, as the positive answers had a percentage of 63.7%, while the negative ones were only 13.0%. In the same way, the percentages of the positive responses "Gave useful recommendations on how to take my medication" were 56.2%, "Made sure I understand how important it is to follow my medication regimen" were 57.2%, and "Worked with me to manage medication-related problems” were 58.2%, respectively. The positive opinions regarding "The pharmacist checked if I understood all the information" were 66.7%, "I could trust the information provided by the pharmacist" were 69.7%, and "The pharmacist spent as much time as necessary to help with my questions and concerns” were 72.2%, which held high rates. Thus, patients are satisfied with the available time that has been spent on them, and they trust the information given. This is consistent with the above assumption that they were not as satisfied as they would have been if they had been given more time to be informed in the overall management of their illness. Additionally, the factor "The pharmacist fully addressed my concerns and health issues during my visit" received a very positive rating of 75.6%, and only 4.5% seem to be dissatisfied with the pharmacist's attitude on this issue. This in turn contributes to the above as we see that the pharmacist addresses the patient's existing concerns, but, at the same time, the patient did not feel that he had received "useful recommendations regarding the management of the overall of health" or had been "encouraged to achieve his therapeutic goals", as he/she would wish. Therefore, the pharmacist tried to fully manage the time given for each patient, but it was not enough. On the other hand, all the above may also be an indication that EOPYY pharmacists should receive additional training on how to manage patient’s concerns and questions in a short and predetermined period, which, in practice, is extremely difficult.

**Table 2:** Detailed listing of all scores Data were presented in the form of n(%).

Factor	Totally disagree	Disagree	I neither agree nor disagree	Agree	Strongly agree
The pharmacist fully addressed my concerns and health issues during my visit	1 (0.5%)	8 (4.0%)	40 (19.9%)	87 (43.3%)	65 (32.3%)
The pharmacist was professional in all our interactions	0 (0%)	5 (2.5%)	27 (13.4%)	87 (43.3%)	82 (40.8%)
The pharmacist gave me information and explained in a way I could understand	1 (0.5%)	7 (3.5%)	30 (14.9%)	86 (42.8%)	77 (38.3%)
The pharmacist checked that I understood all the information	4 (2.0%)	18 (9.0%)	45 (22.4%)	84 (41.8%)	50 (24.9%)
The pharmacist took as much time as necessary to help me with my questions and concerns	3 (1.5%)	14 (7.0%)	39 (19.4%)	87 (43.3%)	58 (28.9%)
The pharmacist made sure I understood how important it is to follow the medication regimen	4 (2.0%)	27 (13.4%)	55 (27.4%)	69 (34.3%)	46 (22.9%)
The pharmacist gave helpful recommendations on how to take my medication	9 (4.5%)	35 (17.4%)	44 (21.9%)	63 (31.3%)	50 (24.9%)
The pharmacist gave helpful recommendations about managing my overall health	11 (5.5%)	43 (21.4%)	63 (31.3%)	55 (27.4%)	29 (14.4%)
The pharmacist worked with me to manage medication-related issues	9 (4.5%)	28 (13.9%)	47 (23.4%)	70 (34.8%)	47 (23.4%)
The pharmacist was caring and kind in dealing with my health issues	2 (1.0%)	10 (5.0%)	33 (16.4%)	92 (45.8%)	64 (31.8%)
The pharmacist encouraged me to achieve my treatment goals	14 (7.0%)	33 (16.4%)	54 (26.9%)	64 (31.8%)	36 (17.9%)
I felt comfortable in my interactions with the pharmacist	2 (1.0%)	13 (6.5%)	27 (13.4%)	103 (51.2%)	56 (27.9%)
The pharmacist was respectful to me during our interactions	1 (0.5%)	7 (3.5%)	22 (10.9%)	105 (52.2%)	66 (32.8%)
The pharmacist is dedicated to improving my health	11 (5.5%)	35 (17.4%)	52 (25.9%)	68 (33.8%)	35 (17.4%)
I could trust the information provided by the pharmacist	7 (3.5%)	11 (5.5%)	43 (21.4%)	85 (42.3%)	55 (27.4%)
I am satisfied with my waiting time	9 (4.5%)	17 (8.5%)	47 (23.4%)	76 (37.8%)	52 (25.9%)
I am satisfied with the information I get	2 (1.0%)	17 (8.5%)	35 (17.4%)	95 (47.3%)	52 (25.9%)
I am satisfied with their professionalism	2 (1.0%)	13 (6.5%)	27 (13.4%)	98 (48.8%)	61 (30.3%)
I am satisfied with their abilities	5 (2.5%)	9 (4.5%)	35 (17.4%)	95 (47.3%)	57 (28.4%)
I am overall satisfied with the EOPYY pharmacy	8 (4.0%)	9 (4.5%)	27 (13.4%)	94 (46.8%)	63 (31.3%)

In Table 3, a bivariate correlation analysis is presented, in which it is observed that all our factors interact with each other at a statistical significance level of 0.01. Then, after establishing that the factors interact with each other, we proceeded to an exploratory factor analysis (EFA) to determine whether these factors correlate to yield variables that can explain with greater coherence and patient's satisfaction with the services provided by EOPYY pharmacies and to identify if any factors should be ignored due to their insignificant contribution.

**Table 3:** Intercorrelations between all variables using Pearson’s correlation analysis ** Level of statistical significance set at 0.01

Variables	1	2	3	4	5	6	7	8	9	10	11	12	13	14	15	16	17	18	19	20
The pharmacist fully addressed my concerns and health issues during my visit	1	-	-	-	-	-	-	-	-	-	-	-	-	-	-	-	-	-	-	-
The pharmacist was professional in all our interactions	0.666**	1	-	-	-	-	-	-	-	-	-	-	-	-	-	-	-	-	-	-
The pharmacist gave me information and explained it in a way I could understand	0.604**	0.685**	1	-	-	-	-	-	-	-	-	-	-	-	-	-	-	-	-	-
The pharmacist checked that I understood all the information	0.537**	0.544**	0.574**	1	-	-	-	-	-	-	-	-	-	-	-	-	-	-	-	-
The pharmacist took as much time as necessary to help me with my questions and concerns	0.623**	0.639**	0.702**	0.630**	1	-	-	-	-	-	-	-	-	-	-	-	-	-	-	-
The pharmacist made sure I understood how important it is to follow the medication regimen	0.479**	0.503**	0.513**	0.595**	0.652**	1	-	-	-	-	-	-	-	-	-	-	-	-	-	-
The pharmacist gave useful advice on how to take my medicines	0.474**	0.479**	0.545**	0.549**	0.547**	0.747**	1	-	-	-	-	-	-	-	-	-	-	-	-	-
The pharmacist gave helpful recommendations about managing my overall health	0.414**	0.393**	0.420**	0.614**	0.516**	0.683**	0.723**	1	-	-	-	-	-	-	-	-	-	-	-	-
The pharmacist worked with me to manage medication-related problems	0.456**	0.476**	0.465**	0.575**	0.582**	0.636**	0.633**	0.740**	1	-	-	-	-	-	-	-	-	-	-	-
The pharmacist was caring and kind in dealing with my health problems	0.511**	0.559**	0.546**	0.445**	0.544**	0.567**	0.584**	0.536**	0.642**	1	-	-	-	-	-	-	-	-	-	-
The pharmacist encouraged me to achieve my treatment goals	0.423**	0.409**	0.412**	0.543**	0.492**	0.600**	0.693**	0.738**	0.717**	0.584**	1	-	-	-	-	-	-	-	-	-
I felt comfortable in my interactions with the pharmacist	0.456**	0.561**	0.503**	0.391**	0.537**	0.460**	0.497**	0.467**	0.528**	0.605**	0.572**	1	-	-	-	-	-	-	-	-
The pharmacist was respectful of me during our interactions	0.520**	0.674**	0.630**	0.455**	0.628**	0.469**	0.528**	0.443**	0.543**	0.652**	0.514**	0.781**	1	-	-	-	-	-	-	-
The pharmacist is dedicated to improving my health	0.460**	0.442**	0.424**	0.573**	0.583**	0.648**	0.685**	0.745**	0.708**	0.625**	0.710**	0.574**	0.581**	1	-	-	-	-	-	-
I could trust the information provided by the pharmacist.	0.479**	0.512**	0.584**	0.530**	0.620**	0.578**	0.636**	0.591**	0.650**	0.550**	0.567**	0.592**	0.681**	0.662**	1	-	-	-	-	-
I am satisfied with my waiting time	0.324**	0.412**	0.291**	0.419**	0.373**	0.348**	0.340**	0.373**	0.338**	0.403**	0.356**	0.436**	0.438**	0.459**	0.405**	1	-	-	-	-
I am satisfied with the information I receive	0.518**	0.598**	0.561**	0.518**	0.631**	0.579**	0.619**	0.534**	0.523**	0.631**	0.526**	0.605**	0.633**	0.626**	0.668**	0.601**	1	-	-	-
I am satisfied with their professionalism	0.514**	0.617**	0.544**	0.437**	0.632**	0.582**	0.528**	0.504**	0.525**	0.639**	0.501**	0.682**	0.706**	0.630**	0.620**	0.514**	0.776**	1	-	-
I am satisfied with their abilities	0.526**	0.604**	0.591**	0.534**	0.679**	0.610**	0.585**	0.551**	0.591**	0.583**	0.539**	0.639**	0.708**	0.704**	0.718**	0.554**	0.745**	0.837**	1	-
I am overall satisfied with EOPYY pharmacy	0.478**	0.498**	0.493**	0.489**	0.535**	0.448**	0.478**	0.477**	0.396**	0.486**	0.374**	0.534**	0.573**	0.541**	0.528**	0.571**	0.677**	0.668**	0.681**	1

We used the maximum likelihood method since we have normal distributions and because it maximizes the differences between the variables and includes an estimate of the relationship created between factors [18], while we used the oblique rotation method as the factors are evaluated for a unique relationship between each factor and the variable [19]. This analysis resulted in the following Table 4, where a total of four variables arose from 18 factors. The factors “I am totally satisfied with my waiting time” and “I am overall satisfied with EOPYY pharmacy” were excluded since, semantically, we considered them to be independent of the others that refer mainly to patient-pharmacist interaction. Factors’ convergence reliability was based also on the EFA where all factors produced communalities greater than 0.5 [20]. Kaiser-Meyer-Olkin (KMO) was 0.947, and Bartlett's test of sphericity significance was 0.000, so our data were suitable for this analysis. In the EFA, we can observe that all factor loadings are above 0.3, and the total variance explained was 77.509%, which is rather adequate [21].

**Table 4:** Exploratory factor analysis summary

Pattern matrix	Factor loadings	Eigenvalue	Var. expl. cumul.
The pharmacist made useful recommendations about managing my overall health	0.992	10.517	60.339
The pharmacist encouraged me to achieve my treatment goals	0.890		
The pharmacist worked with me to manage problems related to medicines	0.793		
The pharmacist is committed to improving my health	0.727		
The pharmacist gave useful recommendations on how to take my medicines	0.721		
The pharmacist made sure I understood how important it is to follow the medication regimen	0.592		
I could trust the information provided by the pharmacist.	0.350		
The pharmacist gave me information and explained it in a way that I could understand	0.874	1.202	68.378
The pharmacist was professional in all our interactions	0.725		
The pharmacist fully addressed my concerns and health issues during my visit	0.698		
The pharmacist took as much time as necessary to help me with my questions and concerns	0.642		
The pharmacist checked that I understood all the information	0.578		
I am satisfied with his professionalism	0.959	0.721	73.963
I am satisfied with his abilities	0.743		
I am satisfied with the information I receive	0.654		
The pharmacist was respectful to me during our interactions	0.769	0.427	77.509
I felt comfortable in my interactions with the pharmacist	0.705		
The pharmacist was caring and kind in dealing with my health problems	0.328		

The variable "medicinal advice", with Cronbach's αlpha coefficient of 0.935, is formed by the following factors: The pharmacist gave helpful recommendations about managing my overall health; the pharmacist encouraged me to achieve my treatment goals; the pharmacist worked with me to manage medication-related issues; the pharmacist is dedicated to improving my health; the pharmacist gave helpful recommendations on how to take my medication; the pharmacist made sure I understood how important it is to follow the medication regimen; and I could trust the information provided by the pharmacist.

Subsequently, the variable "quality of care" was given Cronbach's αlpha coefficient of 0.888, and consists of five individual factors: The pharmacist gave me information and explained it in a way I could understand; the pharmacist was professional in all our interactions; the pharmacist fully addressed my concerns and health issues during my visit; the pharmacist took as much time as necessary to help me with my questions and concerns; and the pharmacist checked that I understood all the information.

Then, the variable "satisfaction with the pharmacist", achieving Cronbach's αlpha coefficient of 0.916, was formed as follows: I am satisfied with their professionalism; I am satisfied with their abilities; and I am satisfied with the information I get.

Finally, the variable "interpersonal relationship" consists of three factors with Cronbach's αlpha coefficient of 0.861: The pharmacist was respectful during our interactions; I felt comfortable in my interactions with the pharmacist; and the pharmacist was caring and kind in dealing with my health issues.

We also tested if our final variables intercorrelate with each other and with the factors “I am totally satisfied with my waiting time” and “I am overall satisfied with EOPYY pharmacy” and tested them to ensure that they have normal distributions. As shown in Table 5, they intercorrelate at a significance level of p = 0.01 (Pearson's correlation bivariate).

**Table 5:** Final constructs' summary statistics and Pearson’s correlation analysis ** Level of statistical significance set at 0.01

Pearson correlation analysis	Final variables	I am overall satisfied with EOPYY pharmacy	I am overall satisfied with EOPYY pharmacy	Interpersonal relationships	Satisfaction with pharmacists	Quality of care	Medicinal advice
	I am overall satisfied with EOPYY pharmacy	1	-	-	-	-	-
	I am satisfied with my waiting time	0.571**	1	-	-	-	-
	Interpersonal relationships	0.598**	0.480**	1	-	-	-
	Satisfaction with pharmacists	0.729**	0.602**	0.788**	1	-	-
	Quality of care	0.598**	0.438**	0.711**	0.733**	1	-
	Medicinal advice	0.544**	0.440**	0.731**	0.742**	0.720**	1
Final constructs summary statistics	Mean			12.144	11.841	20.099	24.622
	Median			12.00	12.00	20.00	26.00
	Std deviation			2.244	2.537	3.659	6.563
	Skewness			-0.778	-0.794	-0.486	-0.337
	Kurtosis			1.096	0.565	-0.177	-0.411

## Discussion

Our findings differentiate from prior studies on Greek public pharmacies [13,14] as we did not capture any availability deficiencies of paramount significance. Additionally, it was rather expected for today’s EOPYY pharmacies to face primarily maturity issues in lieu of the availability ones of the early operation stages. The first key point of our study is that pharmacists were evaluated as moderate regarding the provision of overall health recommendations (e.g., diet, exercise). Actually, this item gathered the lowest mean score (3.24/5.00) among all questionnaire items, suggesting that EOPYY pharmacies are rather focused on their primary role of dispensing drugs and staying away from the “comprehensive care” philosophy [1,2]. This seems like a more generalized failure of the pharmaceutical services since such low satisfaction levels have also been reported by previous studies, also applying the PSPSQ 2.0, on private pharmacies of other health systems [15,22]. Another issue concerns the extent to which pharmacists encourage patients to achieve their treatment goals, gathering the second lowest mean score (3.37/5.00), and therefore reflecting a problematic patient-pharmacist interpersonal relationship. This finding is consistent with previous international research, in accordance with which, patients also provided a moderate to high evaluation [15], while there is another study revealing pure dissatisfaction with this type of encouragement [16]. A third field of unsatisfactory performance hosts the pharmacists’ dedication to improving patients’ health (mean score: 3.40/5.00), which was also found comparatively low in previous studies [15,16,22].

As regards our factor analysis, all three items mentioned in the previous paragraph were loaded in the "medicinal advice" factor, which consequently emerges as the Achilles heel of EOPYY pharmacies. Exactly the same failure has been previously reported for the Japanese community pharmacies, the users of which were found to expect their pharmacists to monitor changes in their lifestyles and provide clear explanations in order to rate them highly as medication specialists [23]. This restricted advisory role may be attributable to time pressure [24], but enhancing the advisory role would reasonably require even more time to be spent on each single patient. In this way, greater time pressure and longer waiting times would occur, and therefore both staff and patients would be rather frustrated with it. Perhaps, this finding reveals the need for structural changes such as adding an advisory/information desk outside the point of drug dispensing, where patients could be informed privately about the health issues concerning them. Such an intervention could increase patient satisfaction in accordance with international experience [25]. In a deeper analysis, the poor advisory performance may also reveal pharmacists’ defensive attitude, albeit justifiable to some extent, owing to chronic and/or severe disease management, against blame and punishment when things go wrong [26]. Hence, pharmacist performance and patient satisfaction could be enhanced by reinforcing patient safety culture [27].

From a different angle, but still concerning the "medicinal advice" deficiency, an English study suggested that most patients prefer to be advised by their family doctor (GP) about medicine-related problems, rather than by the pharmacist, primarily due to poor expectations of pharmacists’ capacity to resolve problems [28]. In response to a “taint” like this, Canadian pharmacists seek practice in family medicine groups (FMGs) toward collaborating with other health professionals and undertaking more clinical and advisory responsibilities [29]. We wonder, then, whether FMG-type schemes are applicable to EOPYY pharmacists. Our initial thoughts are negative given that the EOPYY has lost its vertical integration; namely, all its other healthcare settings and staff have been transferred to the Ministry of Health (now as urban health centers). In short, it would be more difficult for EOPYY pharmacists to practice in interdisciplinary healthcare groups, while public hospitals' pharmacists, who also manage high-cost drugs, retain a clear comparative advantage. Beyond the unusual fact, when compared to international practice, that a social insurance fund, even the central one, dispenses high-cost drugs within its own settings, the operational merge between the EOPYY’s and public hospitals’ pharmacies could be an additional answer to the moderate performance of the former ones in terms of medicinal advice.

Regarding EOPYY pharmacies’ strengths, our findings suggest a relatively high overall satisfaction (mean score: 3.97/5.00), while at the same time, pharmacists’ professionalism prevailed among all questionnaire items (mean score: 4.22/5.00). This signifies not only trust and respect for this type of healthcare unit and its staff but also for pharmacists’ professional capacity to play a vital role in any organizational changes within the pharmacy setting, such as those proposed in the previous paragraphs and others applied internationally [30].

Notwithstanding the several positive implications of our study, there are limitations. First, all respondents had visited pharmacies in Athens; thus, satisfaction with EOPYY pharmacy services in the rest of Greek territory remains unknown. There, the density of EOPYY pharmacies is clearly lower, so there is a possibility that the accessibility problems of the past [12,13] still apply. Second, it should be investigated in further detail how disease severity and current health condition affect satisfaction, given that patients’ needs differ significantly depending on the features above.

## Conclusions

This was the first study to analyze patients’ feelings about EOPYY pharmacies, namely, those public pharmacies operating in the Greek territory by the central health fund and aiming at dispensing primarily high-cost medicines. Our study verified the existing trend that patient expectations from community pharmacies have shifted from simply dispensing prescriptions to a comprehensive care model. More specifically, patients were found to expect more with respect to overall health recommendations, encouragement to achieve their treatment goals, and dedication to improving their health, which consists of a clear message to policymakers and EOPYY’s managers to prioritize strengthening the advisory role of pharmacists. Ultimately, the overall performance of this kind of public pharmacies, as reflected by their users’ satisfaction levels, is deemed satisfactory, and there is potential for further improvement under certain structural and policy interventions.
